# Cardiac Concomitants of Feedback and Prediction Error Processing in Reinforcement Learning

**DOI:** 10.3389/fnins.2017.00598

**Published:** 2017-10-30

**Authors:** Lucas Kastner, Jana Kube, Arno Villringer, Jane Neumann

**Affiliations:** ^1^IFB Adiposity Diseases, Leipzig University Medical Center, Leipzig, Germany; ^2^Max Planck Institute for Human Cognitive and Brain Sciences, Leipzig, Germany; ^3^Faculty 5–Business, Law and Social Sciences, Brandenburg University of Technology Cottbus–Senftenberg, Cottbus, Germany; ^4^Clinic of Cognitive Neurology, University Hospital Leipzig, Leipzig, Germany; ^5^Mind and Brain Institute, Berlin School of Mind and Brain, Humboldt-University, Berlin, Germany; ^6^Department of Medical Engineering and Biotechnology, University of Applied Sciences, Jena, Germany

**Keywords:** reinforcement learning, prediction error, reward, punishment, heart rate, gender, obesity

## Abstract

Successful learning hinges on the evaluation of positive and negative feedback. We assessed differential learning from reward and punishment in a monetary reinforcement learning paradigm, together with cardiac concomitants of positive and negative feedback processing. On the behavioral level, learning from reward resulted in more advantageous behavior than learning from punishment, suggesting a differential impact of reward and punishment on successful feedback-based learning. On the autonomic level, learning and feedback processing were closely mirrored by phasic cardiac responses on a trial-by-trial basis: (1) Negative feedback was accompanied by faster and prolonged heart rate deceleration compared to positive feedback. (2) Cardiac responses shifted from feedback presentation at the beginning of learning to stimulus presentation later on. (3) Most importantly, the strength of phasic cardiac responses to the presentation of feedback correlated with the strength of prediction error signals that alert the learner to the necessity for behavioral adaptation. Considering participants' weight status and gender revealed obesity-related deficits in learning to avoid negative consequences and less consistent behavioral adaptation in women compared to men. In sum, our results provide strong new evidence for the notion that during learning phasic cardiac responses reflect an internal value and feedback monitoring system that is sensitive to the violation of performance-based expectations. Moreover, inter-individual differences in weight status and gender may affect both behavioral and autonomic responses in reinforcement-based learning.

## Introduction

Reinforcement learning describes the process of adapting behavior according to the consequences of actions. Actions or choices that lead to reward or positive feedback should be repeated in similar future situations, whereas actions or choices followed by punishment or negative feedback should be avoided. Thus, in reinforcement learning positive and negative feedback provide the learner with the necessary information for successful behavioral adaptation.

In this paper, we wished to address three research questions: (1) Can we observe systematic differences in leaning from reward and learning from punishment in reinforcement learning? (2) How is feedback processing and learning reflected in phasic cardiac responses during reinforcement learning? (3) How do gender and weight status impact on behavioral measures and cardiac concomitants of reinforcement learning? Thus, we applied an experimental design that comprised independent reward and punishment conditions. During task performance continuous ECG measurements were obtained. Moreover, computational modeling was applied to behavioral and autonomic measures. The paper is structured as follows. We first introduce the main concepts of reinforcement learning and phasic cardiac responses and derive hypotheses for our research questions, followed by the presentation of our experimental task, measurement techniques and analysis methods. We then present our results in the order of our research question, i.e., first regarding the behavioral level, second regarding the autonomic level, and finally regarding the effects of weight status and gender on both behavioral and autonomic measures.

Reinforcement learning in humans has been widely studied in health and disease, and impaired reinforcement learning mechanisms have been identified in various psychiatric and neurological disorders including Parkinson's disease, Huntington's disease, depression, schizophrenia, and several addictive disorders (e.g., de Ruiter et al., 2009; Park et al., 2010; Gradin et al., 2011; Maia and Frank, 2011). Some studies thereby point at differential impairments in learning from reward and learning from punishment (e.g., Frank et al., 2004; Mathar et al., 2017b). In healthy populations, several studies highlight parallels in learning from reward and punishment (Kim et al., 2006; Delgado et al., 2008) including the critical involvement of the brain's dopaminergic system in both learning mechanisms (Glimcher, 2011; Mathar et al., 2017b). However, previous research also identified differences such as, increased reaction times in punishment—compared to reward-based learning, a tendency for reduced learning from punishment, and differential functional brain responses in relation to reward and punishment, sometimes even in the absence of detectable differences in task performance (Delgado et al., 2000; Robinson et al., 2010a; Mattfeld et al., 2011). The involvement of partially different neutrotransmitter systems in reward and punishment processing provides additional evidence for distinct albeit overlapping processing mechanisms for reward and punishment (Guitart-Masip et al., 2014; Jocham et al., 2014). Thus, processing of reward and punishment has to be considered differentially in the investigation of feedback-based learning.

The first goal of our study was a systematic assessment of potential differences in learning from reward and learning from punishment. We employed a probabilistic reinforcement learning paradigm consisting of independent reward and punishment conditions, where learners were provided with only positive and only negative feedback, respectively. Ecological validity of the task and participants' task comprehension were tested by valence and arousal ratings for the presented stimuli prior to and after learning. The overall score achieved at the end of the experiment and the number of participants' advantageous choices and response times were examined as measures of task performance. Participants' choice inconsistency was assessed as measures for behavioral adaptation. Behavioral assessment was complemented by computational modeling that facilitates a deep and detailed analysis of learning on a trial-by-trial basis.

In reinforcement learning, behavioral adaptations are driven by the prediction error (PE) signal (Schönberg et al., 2007). The PE signal encodes the deviations between the expected and the actual outcome of an action. A positive PE arises in situations where an outcome is better than expected, and a negative PE signifies that an outcome is worse. The strength or amplitude of the PE reflects the degree of deviation between expected and actual outcome, whereby fully unexpected and surprising events result in larger PEs. Strength and directionality of the PE signal determine how much and in which direction our current behavior should be adapted for the future. On the neural level, the PE signal is encoded in dopaminergic structures of the midbrain and relayed from there to striatal and prefrontal target regions to drive learning (Schultz et al., 1997; Schultz, 2002).

The construction of PE signals during learning relies on multiple skills starting with the ability to constantly monitor incoming feedback and to correctly build and maintain value representations. Further, value representations have to be updated over the course of learning, and behavior has to be adjusted accordingly for future actions and decisions. Based on behavioral observations alone, these various aspects of the learning process cannot clearly be disentangled. Computational neuroscience provides established mathematical models for reinforcement learning that implement the different aspects of learning and thus facilitate their detailed analysis. When applied to individual behavioral data, these models identify inter-individual differences in learning performance and decision strategies (e.g., Rodriguez et al., 2006; Klein et al., 2007; Lee et al., 2014; Mathar et al., 2017b) and facilitate the estimation of trials-wise PE signals and subject-specific model parameters from the data (Sutton and Barto, 1998; Gläscher and O'Doherty, 2010). Commonly, reinforcement learning models estimate in each trial value representations for the available options. Once an option was chosen by the learner, the PE signal is calculated as the difference between the corresponding value representation and the observed feedback. Value representations are then updated according to the strength and directionality of the PE signal. The most important model parameter in computational reinforcement learning models is the learning rate α. This model parameter is specific for each participant and determines the degree to which value representations are updated after feedback. In other words, the learning rate reflects how strongly new experiences in one trial impact on the participant's knowledge acquired over all previous trials. In addition, most models, including ours, provide a consistency parameter β which reflects how deterministic or stochastic the learner behaves over the course of the experiment. Comparing this parameter to the observed switching behavior of the participant provides a measure for model adequacy, i.e., for how well the computational model captures participants' behavior.

Complementing our behavioral analysis, we fitted for each participant a computational reinforcement learning model to the behavioral data. The consistency parameter was used to ensure model adequacy. Subsequently, trial-wise PE signals and subject-specific learning rates were derived from the model and analyzed to identify the sources of observed behavioral effects.

For our behavioral and computational modeling analysis we derived the following two hypotheses from previous research: **Hypothesis 1:** Across participants, reinforcement learning should be reflected by an increase in correct responses and overall task score as well as a decrease in reaction times over the course of the experiment. **Hypothesis 2:** Learning performance and reaction times were expected to differ between reward and punishment conditions with a potentially reduced performance, smaller model-derived learning rates, and increased reaction times in the punishment condition.

Reactions of the autonomic nervous system provide a further invaluable source of information for the investigation of feedback processing and learning. Numerous previous studies investigated cardiac responses to external stimuli and feedback, taking into account their valence and information content. Concurrently, these studies observed a distinct pattern of phasic heart rate (HR) responses to the presentation of external stimuli: an initial cardiac deceleration that peaks within one second after stimulus onset which is followed by an acceleratory recovery to baseline after 2–4 s. Thereby, it was consistently observed that HR deceleration is prolonged, if the presented stimulus provides negative feedback on a choice or action, in contrast to positive feedback that elicits faster acceleratory recovery (e.g., Crone et al., 2003; Van Der Veen et al., 2004; Groen et al., 2007).

Regarding the information content of a stimulus, experimental results are less conclusive. Van Der Veen et al. (2004) reported prolonged HR deceleration in response to negative feedback which did not discriminate between situations where the feedback was informative or non-informative for the participant. In contrast, Mies et al. (2011) found transient cardiac slowing after negative feedback only in situations where the feedback was valid. In a similar vain, Groen et al. (2007) observed a strong deceleration in response to negative feedback that was prolonged in informative compared to non-informative feedback trials in a probabilistic learning task in children. Importantly, Groen and colleagues also reported a general reduction in feedback-related HR deceleration over the course of learning, and Crone et al. (2004b, 2005) observed HR slowing already in anticipation of feedback, in particular when potentially high gains or losses were to be expected.

Taken together these previous studies suggested that HR deceleration in response to feedback might be caused by a deviation between an expected and an actual outcome of an action (Somsen et al., 2000; Crone et al., 2003). Further, they point at a shift from reliance on external feedback to an internal feedback monitoring system over the course of learning (Crone et al., 2004b; Groen et al., 2007). However, one major caveat of the previous work is that it provides only indirect evidence for these hypotheses, as deviations from performance-based expectations could not be assessed on a trial-by-trials basis. The second goal of our study was to provide direct evidence for a link between trial-wise performance and phasic cardiac responses during learning and feedback processing. Specifically, the use of the computational model enabled us to directly correlate the strength of PE signals with the strength of autonomic responses.

From the presented previous observations, we derived the following additional hypotheses for our experiment: **Hypothesis 3:** We expected a significant HR deceleration in response to feedback presentation which is more pronounced for negative than for positive feedback. **Hypothesis 4:** Over the course of learning, HR responses should shift from the presentation of feedback toward the anticipation of potential feedback already at the time of stimulus presentation. **Hypothesis 5:** The strength of phasic HR responses should directly predict the strength of PE signals on a trial-by-trial basis.

In our previous research, we identified weight status and gender as important interacting factors influencing feedback processing and reinforcement learning on the behavioral and neural level (e.g., Horstmann et al., 2011; García-García et al., 2014; Mathar et al., 2017a; Kube et al., submitted). In the context of obesity, this might be explained by profound alterations of the brain's dopaminergic system (Wang et al., 2001; de Weijer et al., 2011; Volkow et al., 2011; Horstmann et al., 2015b) which underlies the coding of PE signals. This goes along with wide-spread obesity-related changes in both brain structure and function which extend from striatal regions into sensory and cognitive control-related frontal cortices implied in outcome processing and reinforcement learning (Horstmann et al., 2011; Kullmann et al., 2011; García-García et al., 2015; Figley et al., 2016; Hogenkamp et al., 2016).

The rewarding properties of food and increased responsivity to food cues in obesity have been widely studied (e.g., Stice et al., 2009; García-García et al., 2014; Pursey et al., 2014; Alonso-Alonso et al., 2015; Horstmann et al., 2015a; Mathar et al., 2016; Mühlberg et al., 2016). In contrast, the differential processing of reward and punishment and reinforcement learning in a none-food context are far less understood in individuals with obesity. Coppin et al. (2014) presented first evidence for performance deficits in a probabilistic reinforcement learning tasks in individuals with obesity along with working memory differences between lean and obese participants. Importantly, obesity-related deficits in reinforcement learning were specific to the avoidance of negative outcomes suggesting a differential sensitivity to positive and negative feedback. Opel et al. (2015) reported increased neural responses in reward-related brain regions in individuals with obesity when presented with monetary gains, with no obesity-specific alterations in the processing of losses. In contrast, Balodis et al. (2013) observed greater functional activation in subcortical and prefrontal brain regions in individuals with obesity for the processing of both monetary gains and losses. Thus, evidence for obesity-specific deficits in reinforcement learning and differential processing of reward and punishment in obesity is still inconclusive.

Gender-related influences on feedback processing and learning have likewise been reported in previous studies. For example, higher performance levels in men than in women were observed in reversal learning and the well-known Iowa Gambling task (Weller et al., 2009; Evans and Hampson, 2015). Robinson et al. (2010b) report gender effects of dopamine depletion on learning from punishment with significant improvement of punishment processing after dopamine depletion in women, but not in men. In addition, in the context of feedback processing and learning gender was found to closely interact with obesity. For example, women with obesity showed a preference for risky choices despite infrequent punishment with high penalties as well as decreased behavioral adaptation after punishment in the Iowa Gambling Task (Horstmann et al., 2011). Interestingly, this was accompanied by gender-specific correlations between markers of obesity and gray matter volume (GMV) in brain structures involved in learning, cognitive control, and goal-directed behavior.

The impact of weight status and gender on general heart rate variability (HRV) have long been known (e.g., Zahorska-Markiewicz et al., 1993; Ramaekers et al., 1998; Karason et al., 1999; Windham et al., 2012; Koenig and Thayer, 2016). However, to the best of our knowledge only two studies from our own lab assessed phasic HR changes in the context of gender and obesity to date. In these studies we observed, for obese women specifically, blunted cardiac responses to social compared to monetary stimuli (Kube et al., 2016) together with strong cardiac slowing in novel social interactions (Schrimpf et al., 2017).

The third goal of our study was to explore the effects of weight status and gender on phasic HR changes in the differential processing of reward and punishment during reinforcement learning. Further we aimed at consolidating the heterogeneous previous findings on the behavioral level by a systematic assessment of obesity—and gender-specific alterations in learning performance, behavioral adaptation and computational model parameters. We expected weight status and gender to impact on both performance and HR responses, possibly differentially for reward and punishment. However, sparsity and inconsistency of previous results, as shown above, precluded clear hypotheses for the size and direction of these effects, rendering the present assessment of these two factors more exploratory.

Finally, we have to consider different personal characteristics that might influence reinforcement learning from reward and punishment. Participants' general sensitivity to reward and punishment may impact on reinforcement learning performance, given that the learning process heavily relies on the adequate evaluation of rewarding and punishing feedback. In addition, weaknesses in learning from reward and punishment and impaired adaptation of choice behavior have previously been linked to high trait impulsivity (e.g. Franken et al., 2008), although an earlier study by the same authors did not result in conclusive evidence for this relationship (Franken and Muris, 2005). In the same vein, it was argued that working memory capacity crucially impacts on reinforcement learning (Collins and Frank, 2012), in particular on the processing of PE signals (Collins et al., 2017). Therefore, participants' working memory capacity, reward and punishment sensitivity, and trait impulsivity were taken into account in our analyses.

## Materials and methods

### Participants

Sixty Caucasian participants, aged between 18 and 36 years, were initially invited to our experiment. All participants were right-handed, had normal or corrected-to-normal vision, and were grouped according to BMI into a group of participants with (BMI ≥30 kg/m^2^, <45 kg/m^2^) and without (BMI ≥18.5 kg/m^2^, <25 kg/m^2^) obesity. Participants were recruited from the participant database of the Max Planck Institute for Human Cognitive and Brain Sciences, Leipzig, Germany. All participants provided written informed consent prior to participation. The study complies with the ethical standards of the Declaration of Helsinki and was approved by the ethics committee of the University of Leipzig.

All participants underwent an initial telephone screening to evaluate inclusion and exclusion criteria. Exclusion criteria were a history of neurological or neuropsychiatric disorders, current smoking, recent or current dieting, use of drugs, psychoactive medication, or medication influencing the autonomic nervous system. These exclusion criteria were chosen to avoid confounding alterations in reinforcement processing due neuropsychiatric symptomatology and medication (Etkin and Wager, 2007; Wittmann and D'Esposito, 2015), smoking status (Martin et al., 2014), and hunger (Symmonds et al., 2010; Levy et al., 2013). Participants reporting hyper- or hypothyroidism were excluded, since these conditions may affect their baseline cardiac responses as well as body weight status (Bratusch-Marrain et al., 1978; Cacciatori et al., 2000; Tzotzas et al., 2000). As previous studies have shown that hypertension may be associated with altered baseline cardiac responses (Schroeder et al., 2003; Kim et al., 2016), we excluded participants who reported hypertension during the telephone screening or exhibited values exceeding the range for normal or high normal blood pressure (Mancia et al., 2013) in a manual examination after the experiment. Further, a depressive symptomatology has been found to be associated with altered HR responses to the presentation of rewarding stimuli (Brinkmann and Franzen, 2013, 2017). Therefore, we measured the current depressive symptomatology using Beck's Depression Inventory-Short Form (BDI-SF, Beck and Steer, 1993) and excluded participants with a BDI-SF > 10. Finally, as even moderate physical exercise impacts on measures of HR and HRV (Rennie et al., 2003; Hottenrott et al., 2006), we excluded participants with more than 3 h per week of regular cardiovascular training.

Upon participation, a total of 12 participants had to be excluded due to an excessive number of miss trials during the experiment (5), insufficient task comprehension identified during a debriefing interview (6) and technical problems (1). Thus, our final sample consisted of 48 participants (mean age: 25.9 ± 4.37 years; range between 20 and 36 years) including 24 participants with obesity (BMI = 35.59 kg/m^2^ ± 3.39 kg/m^2^, range 30.68–43.33 kg/m^2^, 12 female) and 24 lean participants (BMI = 22.18 kg/m^2^ ± 1.37 kg/m^2^, range 19.83–24.09 kg/m^2^, 12 female). Groups were matched for age and level of educational background. For the latter we chose years of scholastic education as a comparable objective variable. All but two participants finished at least 12 years of scholastic education, which in the German educational system is the prerequisite to enter university to receive higher education. Two participants finished secondary school after 10 years followed by vocational training, which represents the second highest level of scholastic education.

### Experimental task

**Figure 1:**
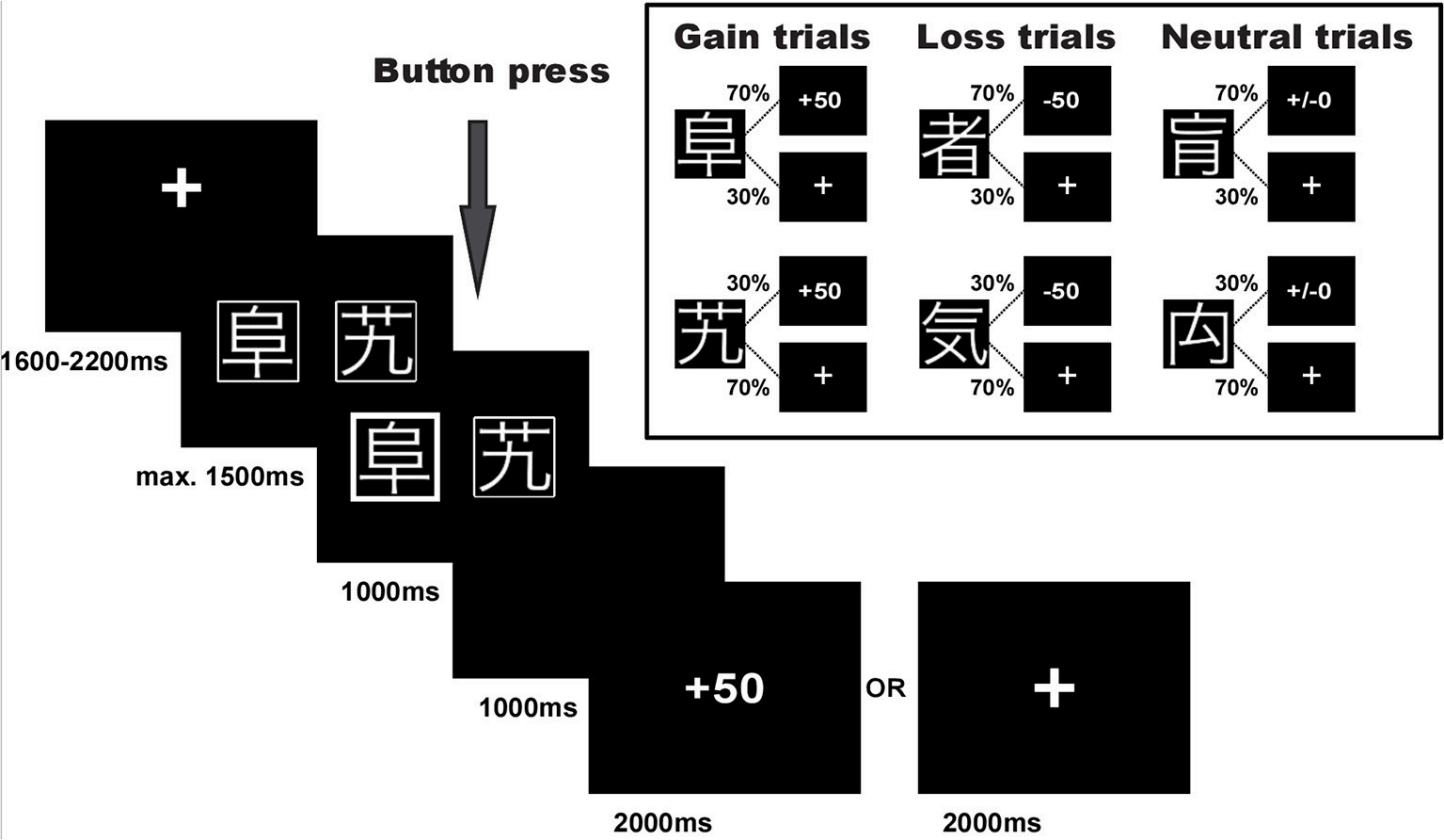
Experimental task. Example trial and task structure with reward/gain and punishment/loss probabilities of the reinforcement learning task.

Participants performed a probabilistic reinforcement learning task adapted from Kim et al. (2006) and Bódi et al. (2009). The task consisted of 240 trials. In each trial participants were presented with a pair of symbols and had to choose one of them by button press. Three different pairs of symbols were included in the experiment: (1) one pair signaled the possibility of winning 50 points or receiving no outcome (80 reward/gain trials), (2) one pair signaled the possibility of losing 50 points or receiving no outcome (80 punishment/loss trials), and (3) one pair was associated with a neutral outcome signaling neither gain nor loss (80 neutral trials), see Figure 1. In each pair, one symbol was associated with a higher probability of receiving the respective outcome: In gain trials, the advantageous symbol was associated with a 70% probability of winning 50 points and lead to no outcome in only 30% of the trials in which it was chosen. The disadvantageous symbol was associated with only a 30% probability of winning 50 points and led to no outcome in 70% of the trials in which it was chosen. Similarly, in loss trials the advantageous symbol was associated with a 70% probability of avoiding to lose 50 points, while the other symbol had a loss avoidance probability of only 30%. In the neutral control condition, the two symbols likewise had a 70 and 30% probability of seeing neutral feedback, and 30 and 70% probability of no outcome, respectively. Symbols were randomly assigned to a given trial type, and trial order was randomized in blocks of 30 trials to ensure a roughly equal number of trials per condition in each stage of the experiment.

Note that with this task design, participants could maximize their overall task performance by choosing the advantageous symbol in both the reward and the punishment condition, i.e., by learning to select the high probability reward symbol in reward trials and the high probability punishment avoidance symbol in punishment trials. Choices during neutral trials did not affect task performance. Importantly, independence of positive and negative feedback in this task design additionally enabled us to determine individual differences in learning from the two feedback valences.

Trial timing in an example trial for the reward condition is displayed in Figure 1. The pair of stimulus symbols was presented for a maximum of 1,500 ms and participants were asked to select one of them. Once the symbol was selected, the chosen option was highlighted for 1,000 ms and a blank screen followed for a 1,000 ms delay period. Thereafter, the outcome was presented for 2,000 ms. If the participants received no outcome, a fixation cross was shown instead. If the participants did not press the button or were too slow, the trial was aborted and the text “zu langsam!” (too slow) appeared on the screen. These trials were dismissed from further analyses (1.5% of all trials). Each trial was followed by an inter-trial-interval of 1,600–2,200 ms.

Prior to the experiment, participants were instructed about the task and performed a practice run of 12 trials, four trials in each condition. The instructions included the information that two symbols would be presented in each trial and the task was to select one of them. Depending on their choice participants would win 50 points, lose 50 points, receive a financially neutral outcome or no feedback. Participants were informed that the task comprised of three trial conditions and that in each trial condition one symbol had a higher probability of leading to an advantageous outcome. However, they did not know which symbol was associated with a particular outcome. In addition, participants were informed that their net gain would be transformed into a monetary bonus at the end of the experiment. Upon completion of all tasks and questionnaires participants were debriefed about the aim of the study.

### Experimental procedure

Experiments were performed in a sound proof room that was artificially lid with the blinds closed. Upon arrival, participants were explained the procedure and comfortably seated on a chair in front of a computer screen. They first performed the working memory test. Participants were then prepared for electrocardiogram (ECG) recording. To allow ECG to stabilize after preparation, participants filled in the first questionnaire. This was followed by a 5 min baseline recording of ECG data and the experimental task, including pre- and post-ratings of the symbols. After the experimental task was finished, participants filled out a second set of questionnaires, and were debriefed about the experiment. Finally, participants' current height, weight, and blood pressure were measured. The entire experiment lasted for ~2 h. Participants received reimbursement of 7 Euro per hour and additional bonus of 2.86 Euro on average, according to the score reached by the end of the experiment.

### Data acquisition

#### ECG data

The ECG was continuously recorded during the task with a sampling rate of 500 Hz using BioPac 3.7.7 and the MP35 recording unit. In order to ensure that participants show typical and healthy cardiac responses at rest, ECG was also recorded for HRV analysis during a 5 min resting period before the start of the experiment. Three Ag/AgCl ECG electrodes (Nessler Swaromed) were placed below the right collar bone about 10 cm from the sternum, on the left side between the lower two ribs, and on the right lower abdomen. ECG data analysis was carried out using customized Matlab-based scripts (Matlab R2013b, The MathWorks, Sherborn, MA, USA) for R-peak detection and artifact correction. Automatic R-peak detection identified all stationary data points that exceeded 20% of the global ECG maximum and were preceded by data points with a first derivative 1.5 times larger than the global ECG maximum. A median template of the QRS-complex around the detected R-peaks was calculated, and QRS complexes with a cross correlation coefficient larger than 0.8 were selected. All automatically detected R-peaks were visually inspected to ensure correct R-peak detection and manually corrected where necessary. Inter-beat-intervals (IBI) were calculated as time difference between two subsequent R-peaks. IBIs deviating more than 3.5 SDs from the session's mean IBI or more than 50% from the preceding IBI were identified as artifacts and replaced by the session's mean IBI length. Note that this approach differs from the often applied interpolation by neighboring IBIs. However, we avoided any interpolation from neighboring IBIs in ECG data modeling, as the statistical analysis of phasic IBI changes crucially depends on the direct comparison of neighboring IBIs. Interpolating corrupted IBIs by their neighbors might therefore compromise the validity of the statistical analysis. Across participants only 0.37% of all IBIs (421 out of 112,016) were identified as artifacts with an average of 0.36% of IBIs per person. The largest number of IBIs replaced for an individual participant amounted to 66 out of 3,034. These very few artifacts were unlikely to significantly impact on subsequent statistical analyses.

#### Personality traits, working memory scores, and ratings

Four potential influencing factors were regarded in the behavioral analysis and assessed for each participant prior to or after the task: participants' responsiveness to reward, responsiveness to punishment, impulsivity, and working memory capacity. The first three factors were assessed by means of two questionnaires, the BIS/BAS (Carver and White, 1994) and the UPPS Impulsive Behavior Scale (Whiteside and Lynam, 2001). The BIS/BAS captures two general motivational systems that underlie behavior. The Behavioral Inhibition System (BIS) represents an aversive motivational system that is sensitive to punishment and reward omission. The Behavioral Activation System (BAS) reflects an appetitive motivational system which is sensitive to reward and the avoidance of punishment. Note that the internal consistency of the three BAS factors drive, fun seeking, and reward responsiveness is still under debate for the German version of the questionnaire that was used in our experiment (Strobel et al., 2001; Mueller et al., 2013). Observed effects regarding these factors should thus be treated with caution.

The UPPS is designed to assess distinct personality facets associated with impulsive behavior: urgency, (lack of) premeditation, (lack of) perseverance, and sensation seeking. These four subscales possess very good internal consistency in the German version of the UPPS (Schmidt et al., 2008).

Possible inter-individual performance differences due to visual working memory capacity, were assessed in the German version of the revised Wechsler Memory Scale (WMS-R), subtest Figural Memory (Wechsler, 1987; Härting et al., 2000).

Immediately before and after the learning task, we obtained subjective valence and arousal ratings for each symbol to determine changes in affective responses toward the stimuli. Here, each cue was presented individually and rated according to valence and arousal on 9-point Self-Assessment Manikin visual analog scales (Bradley and Lang, 1994). This enabled us to investigate task-induced differential changes in the evaluation of advantageous and disadvantageous symbols.

### Data analysis

#### Computational model of learning behavior

Trial-wise PEs, subject- and condition-specific learning rates and choice consistency estimates were derived from a computational reinforcement model. The model is an implementation of the Q-learning algorithm (Watkins and Dayan, 1992). It was previously applied in a comparable implicit learning paradigm in healthy individuals and clinical populations, where it was shown to adequately capture reinforcement learning tasks based on time-invariant probabilistic stimulus-outcome associations (Mathar et al., 2017b). In more detail, the model consists of six input nodes *I*_*i*=1,…,6_ with weighted connections to two output nodes (*Q*-values) *Q*_*j*=1,2_ that represent the presence or absence of the six possible symbols *i* (three pairs of symbols) and the two possible outcomes *j* in each condition, respectively. On each trial, activity of the output nodes is computed as

(1) $$\begin{array}{r} Q_{j}=\;\sum_{i}w_{ij}\;I_{i}, \end{array}$$

where *w*_*ij*_ represents the weight connecting input node *I*_*i*_ and output node *Q*_*j*_. Weights are initialized to 0.25, representing equal distribution of initial weights between the four connections that can be updated within one trial (connections from two stimulus symbols at a time to the two outcomes). Weights are updated in each trial *k* by means of

(2) $$\begin{array}{r} w_{ij}\left(k+1\right)=w_{ij}\left(k\right)+\alpha^{r/p/n}S_{j}\left(R_{j}-Q_{j}\right)I_{i}, \end{array}$$

where *R*_*j*_ encodes the actual outcome in this trial and *S*_*j*_ represents the participant's choice. The latter is included for allowing the model to simulate the behavior of the individual participant rather than optimal learning.

To differentially assess learning from reward (potential gains) and punishment (potential losses), we fitted three independent learning rates for the reward α^*r*^, punishment α^*p*^, and neutral condition α^*n*^, respectively. In reinforcement learning, a learning rate reflects how strongly new experiences in one trial impact on the participant's knowledge acquired over all previous trials. For each participant, the three individual learning rates were determined that minimized the sum of squared differences between the model's output and the participant's choice:

(3) $$\begin{array}{r} \sum_{jk}{\left(S_{jk}-Q_{jk}\right)}^{2}\to min, \end{array}$$

with *j* = 1, 2 and *k* again marking the trial number. In a subsequent step, we modeled the probability for each participant's choices of a particular symbol to follow a softmax distribution:

(4) $$\begin{array}{r} P\left(choice=S_{j}|Q_{1},Q_{2}\right)=\frac{exp\left(\beta Q_{j}\right)}{exp\left(\beta Q_{1}\right)+exp\left(\beta Q_{2}\right)}with\;j=1,\;2, \end{array}$$

where the parameter β reflects the consistency of choices made by the participant. That is, the parameter reflects how deterministic or stochastic the participant behaves over the course of the experiment, with high β-values representing more stochastic or inconsistent behavior.

Model fitting and estimation of all parameters was accomplished by non-linear optimization. Recall that the PE in each trial encodes the discrepancy between expected and actual outcome. Thus, after model fitting the prediction error *PE*_*k*_ for trial *k* can be directly derived from Equation (2) as

(5) $$\begin{array}{r} PE_{k}=S_{jk}\left(R_{jk}-Q_{jk}\right)I_{ik}. \end{array}$$

Prior to statistical analysis of model parameters, model adequacy was assessed in two ways. First, the model's choice consistency parameter β was regressed against the overall number of switches between choices exhibited by the participant. A strong regression signifies that, across subjects, the model adequately captured participants' behavior, because if the model correctly reproduces participants' actual behavior, then a model's choice consistency (small β) should go along with few switches made by a participant, while inconsistent choice behavior of the model (i.e., large β) should entail large number of switches by the participant. Second, model fit was compared across participant groups by means of the Bayesian Information Criterion (BIC, Schwarz, 1978), as comparable model fit is a prerequisite for parameter comparability.

#### Autonomic responses

In our event-related HR analysis we closely followed the procedures applied in previous assessments of phasic cardiac concomitants of stimulus and feedback processing (e.g., Somsen et al., 2000; Crone et al., 2003; Van Der Veen et al., 2004; Groen et al., 2007). For the event-related analysis of stimulus processing, four IBIs were extracted around stimulus presentation: IBI 0 was measured at the time of stimulus presentation and was preceded by IBI −1 and immediately followed by IBIs 1 and 2. All stimulus-related IBIs were referenced to a statistically independent IBI −2 prior to trial start. Statistical analyses of this reference IBI revealed no significant valence, gender, or obesity effect (repeated measures ANOVAs with within-subject factor valence (reward, punishment, neutral) and between-subject factors gender and obesity; all *p* > 0.282).

For the event-related analysis of feedback processing, five IBIs were extracted: IBI 0 was measured at the time of feedback presentation and was preceded by IBI −1 and immediately followed by IBIs 1, 2, and 3. In order to marginalize the impact of differential stimulus processing on the feedback-related analysis, all IBIs were now referenced to the IBI −2 prior to feedback presentation. This ensures independence of IBI changes at feedback presentation from IBI changes at stimulus presentation, as a reference IBI after stimulus presentation effectively functions as a new “baseline” preceding feedback presentation. Note that for the purpose of plotting responses to stimuli and feedback on a common scale in **Figure 3**, in this plot all IBIs from stimulus presentation to HR recovery after feedback presentation are referenced to a common IBI-2 prior to stimulus presentation and named IBI 0 (presentation of stimulus) to IBI 7.

In order to assess learning-induced effects on performance over the course of the experiment, experimental trials were divided into four task blocks of 60 trials each. Learning-induced effects on phasic IBI changes were expected to emerge later than behavioral adaptation. They were thus assessed by comparison of the first and the second experimental half, containing trials 1 to 120 and trials 121–240, respectively.

Finally, a potential learning-induced shift in heart beat responsiveness from the presentation of feedback to the presentation of stimuli was directly investigated based on the mean area under the curve (AUC) that describes changes in IBI length following stimulus and feedback presentation. Specifically, for each subject, a trapezoid was calculated, representing the AUC of changes in IBI length from IBI 0 to IBI 2 after the presentation of a stimulus and the presentation of feedback, respectively. Note that reference IBIs for stimulus-related and feedback-related IBIs were identical to the independent analyses of stimulus and feedback processing in order to ensure comparability of AUCs across the two event types. Mean AUCs were then submitted to a repeated measures ANOVA containing event type (stimulus, feedback), experimental half (1st half, 2nd half), and valence (reward, punishment) as within-subject and gender and obesity as between-subject factors.

#### Relationship of the PE signal and autonomic responses

Subsequent to modeling each participant's learning behavior, we analyzed the relationship between the obtained trial-wise PEs and the relative IBI length following feedback presentation. For this, we employed a general linear model (GLM) and subsequent statistical evaluation of the GLM parameters. Specifically, for each subject, the vector of PEs after positive and after negative feedback was modeled as

$$\begin{array}{l} PE_{k}=b_{c}+\;b_{0}IBI0_{k}+\;b_{1}IBI1_{k}+\;b_{2}IBI2_{k}+\;\varepsilon_{k} \end{array}$$

for all trials *k* that led to positive or negative feedback respectively, with the PE derived from the computational learning model as dependent variable, IBI 0, IBI 1, and IBI 2 as independent variables, the corresponding vector of coefficients *b* and the error vector ε. Across subjects, coefficient estimates corresponding to IBI 0, IBI 1 and IBI 2 were subjected to a one sample *T*-test. Coefficients with a significant difference from zero mark a predictive effect of the corresponding independent variable on the size of the PE. In other words, across participants, PE and IBI are systematically associated for any IBI with a GLM coefficient that significantly differs from zero. In order to compare predictive effects across the three IBIs as well as across conditions, GLM coefficients were subsequently subjected to a repeated measures ANOVA with IBI (IBI 0, IBI 1, IBI 2) and valence (reward, punishment) as within-subject factors. For the comparison across IBIs, standardized coefficients were used.

### Statistical methods

All acquired and modeled data as well as their hypothesized interdependencies were statistically analyzed using IBM SPSS Statistics 22.0 (IBM Corp., Armonk, NY, USA). For all statistical tests we assume statistical significance for *p* < 0.05. For each analysis, statistical tests were chosen depending on the nature and distribution of the data as follows. Group differences (lean vs. obese, female vs. male) for normally distributed data in demographics, questionnaire scores, performance measures, BIC-values, and model parameters were analyzed by univariate ANOVAs with obesity and gender as fixed between-subject factors. For normally distributed data we report mean and standard deviation. Mann-Whitney-U-Tests were applied when the assumption of normality was violated as assessed by Shapiro-Wilk test. Here, we report medians and [min, max] of the data or, in cases where the full range of possible values was covered by the results, [25th, 75th percentiles]. Pairwise *post-hoc* comparisons were calculated to assess origin and directionality of interaction effects observed in univariate or repeated measures analyses of variance.

Across participants, performance differences were compared between experimental conditions and between task blocks by related samples Friedman's Two-Way Analysis of Variance by Ranks for three or more conditions or task blocks, and by Wilcoxon signed rank tests for two conditions, respectively. Differences between conditions in the number of switches were statistically assessed by a sign test, as the assumption of the Wilcoxon signed rank test for a symmetrically shaped distribution of differences was not met. Reaction times were analyzed by repeated measures ANCOVA with between-subject factors obesity and gender, within-subject factor valence (reward, punishment, neutral) and task block (blocks 1–4). Age was included as covariate of no interest.

Differences in valence and arousal ratings between symbols prior to the task were assessed by repeated measures ANOVAs with symbol as within-subject factor and obesity and gender as between-subject factors. Task induced changes in valence and arousal ratings were analyzed by repeated measures ANOVAs with time point (pre/post task) as within-subject factor and obesity and gender as between-subject factors. Bivariate correlations between questionnaire scores and working memory capacity with performance measures were determined by Pearson's correlation coefficients. Normality of the data was ensured by Shapiro-Wilk test.

Differences in phasic IBI were statistically evaluated by repeated measures ANOVAs. Specifically, mean IBI differences in response to stimulus presentation were statistically evaluated using a repeated measures ANOVA with valence (reward, punishment), experimental half (1st half, 2nd half), and IBI (four levels; IBI −1, IBI 0, IBI 1, IBI 2) as within-subjects factors and obesity and gender as between-subjects factors. Note that the “factor experimental half” was included in the ANOVAs to identify autonomic reactions that might only be present at the beginning or toward the end of learning. The specific analysis of a potential learning-induced shift in autonomic responsiveness is described below. For the analysis of IBI differences in response to feedback, the within-subject factor IBI consisted of five levels: IBI −1, IBI 0, IBI 1, IBI 2, IBI 3. To identify the underlying cause in IBI differences, e.g., differences in deceleration or recovery speed between conditions or participant groups, changes between neighboring IBIs were assessed. In other words, differences between IBI −1 and IBI 0, between IBI 0 and IBI 1 and so forth were calculated and statistically compared across any interacting effect e.g., between genders or positive and negative feedback trials. Graphically, this is reflected in the steepness of the slope between two neighboring IBIs.

All pairwise *post-hoc* tests and all statistical tests involving dependent data were Bonferroni corrected for multiple comparisons. The latter included, for example, all tests involving the number of switches, all correlations of the same performance measure with personality traits etc. In cases where correction for multiple comparisons was required, we report only those *p*-values as significant that are below the adjusted significance threshold and report the applied number of tests as correction factor (CF), e.g., *p*-values below the adjusted threshold of *p* < 0.025 and the correction factor CF 2 in case of two tests on dependent data.

## Results

### Demographics

Descriptive statistics of participants' demographic characteristics are reported in Table 1. As intended, across groups participants did not differ with respect to age and educational background. Lean and obese participants significantly differed in weight, BMI, and waist-to-hip ratio. Male and female subjects significantly differed in height, weight, and waist-to-hip ratio but, importantly, not in BMI distribution. Gender and weight status can thus be regarded as independent factors in the statistical analysis. In the same vein, baseline HR did not significantly differ between groups and HRV analysis at rest revealed typical patterns of cardiac activity (Supplementary Material), ruling out any impact of those factors on the subsequently observed effects in phasic cardiac responses.

**Table 1:** Descriptive statistics.

	**LEAN**		**OBESE**		***p***	
	**Male**	**Female**	**Male**	**Female**	**Factor obesity**	**Factor gender**
Age (years)	26.2 (5.78)	25.0 (4.41)	26.7 (3.2)	26.0 (4.11)	0.564	0.482
Years of education	13 (13-13)	13 (13-13)	13 (10-13)	13 (10-13)	0.187	0.657
Height (m)	1.80 (0.04)	1.71 (0.06)	1.80 (0.7)	1.67 (0.06)	0.206	**<0.001**
Weight (kg)	73.37 (4.77)	63.75 (6.57)	115.24 (15.54)	99.37 (9.83)	**<0.001**	**<0.001**
BMI	22.63 (1.20)	21.73 (1.44)	35.59 (3.24)	35.61 (3.68)	**<0.001**	0.565
WHR (cm)	0.82 (0.04)	0.75 (0.04)	0.95 (0.05)	0.84 (0.05)	**<0.001**	**<0.001**
HR (beats per min)	66.50 (8.74)	65.33 (7.5)	65.17 (10.53)	67.17 (9.78)	0.925	0.876

*Distribution of gender, age, level of education, height, weight, body mass index (BMI), waist-to-hip ratio (WHR), and baseline heart rate in male and female participants with and without obesity. Values represent mean and standard deviation except for years of education [median (min-max)]. Group differences were determined by univariate ANOVA with obesity and gender as fixed between-subject factors. Significant group effects at p < 0.05 are marked in bold*.

### Behavioral analysis

Addressing hypotheses 1 and 2, we first analyzed participants' task performance in the reinforcement learning task according to the overall score achieved and according to the number of advantageous choices made over the course of the experiment. In addition, we defined a learning criterion for the reward and punishment condition that allowed us to assess speed of learning as follows: A participant had successfully learned the task, if he or she chose the symbol with high probability of receiving a reward and with high probability of avoiding a punishment in 9 out of 10 consecutive trials in the reward and punishment condition, respectively.

**Figure 2:**
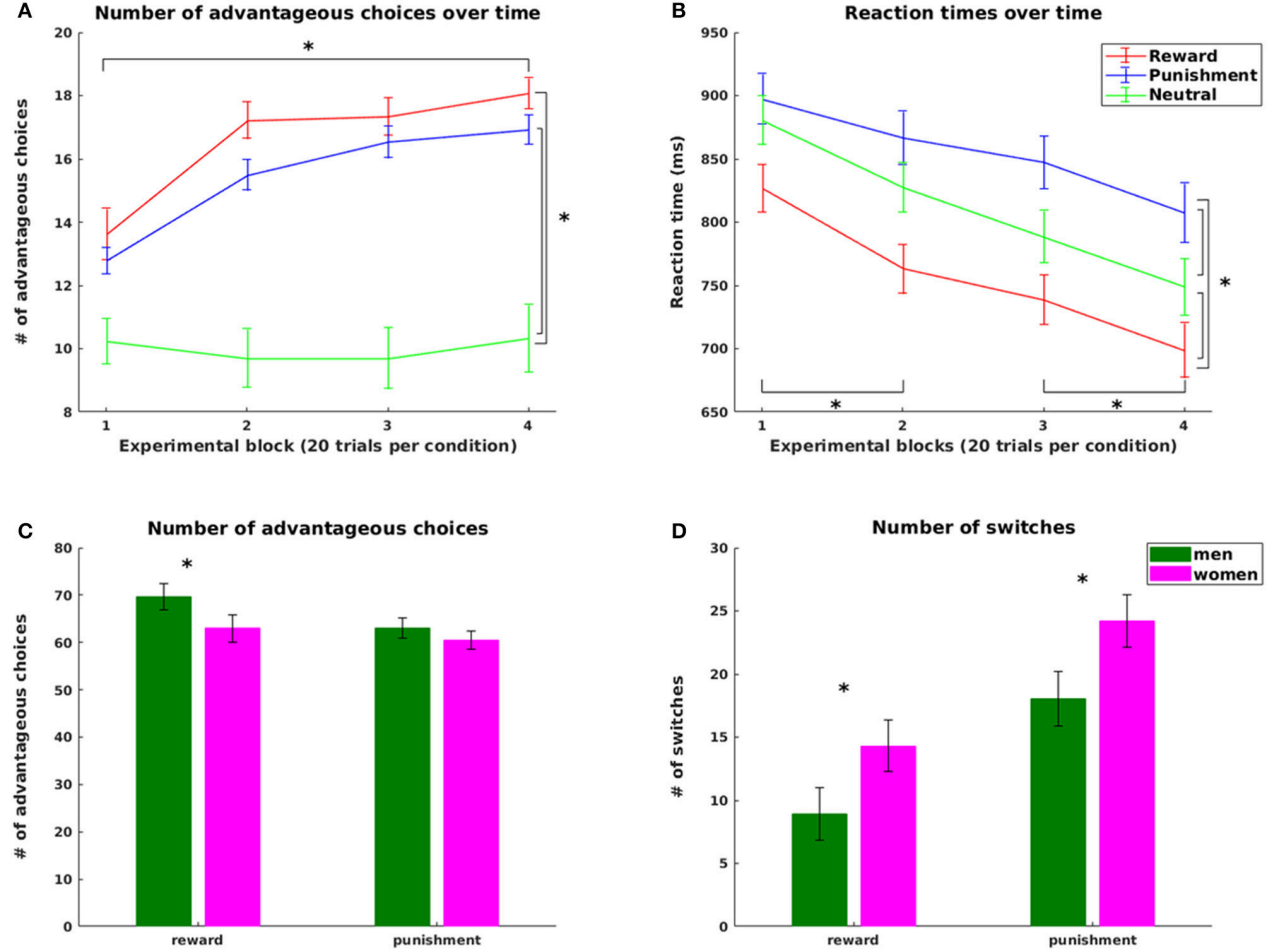
Behavioral results. Top: Number of advantageous choices **(A)** and reaction times **(B)** for the reward, punishment and neutral condition; bottom: Significant gender effects in the number of advantageous choices **(C)** and in the overall number of switch **(D)** trials for the reward and punishment condition. Advantageous choices refer to trials where participants chose the symbol with the higher probability for gaining a reward or avoiding a punishment in the reward and punishment condition, respectively. Switch trials refers to those trials where participants changed their choices from one symbol in the previous trial of this condition to the other symbol in the current trial. Statistically significant differences at *p* < 0.05 are marked with ^*^.

Supporting hypothesis 1, all participants increased their scores from the initial 2,000 points with final scores ranging from 2,150 to 3,550 points. The number of advantageous choices significantly increased over the four experimental blocks for the reward and the punishment condition [choose reward: $\chi_{\left(3\right)}^{2}$ = 53.975, *p* < 0.0005; avoid punishment $\chi_{\left(3\right)}^{2}$ = 50.940, *p* < 0.0005, Figure 2A]. No difference across blocks was observed in the neutral condition (*p* = 0.49). Both number of advantageous reward and punishment choices were significantly higher than the number of neutral choices with high probability feedback (reward: z = 5.32, *p* < 0.0001; punishment z = 5.03, *p* < 0.0001), pointing at successful learning from both reward and punishment. However, in line with our hypothesis 2, the number of advantageous choices was significantly higher in reward compared to punishment trials (z = 2.470, *p* = 0.014), reflecting an increased influence of positive compared to negative reinforcement during learning.

Adding to the differences in task performance across conditions, we observed a statistically significant difference in the time point of reaching the learning criterion: Participants reached the learning criterion on average after 14 [25th and 75th percentile: (10,26)] reward trials, but only after 23 [25th and 75th percentile: (15,35)] punishment trials (z = −1.98, *p* = 0.047). Note that four participants did not reach the learning criterion in one or both conditions. These four participants were excluded from all analysis involving the learning criterion.

Performance scores and number of advantageous choices significantly correlated in a negative way with the overall number of switches in both reward (score *r* = −0.787, advantageous choices: *r* = −0.831) and punishment trials (score: *r* = −0.639, advantageous choices: *r* = −0.870, all *p* < 0.005, CF 4). Importantly, both the number of switches before and after reaching the criterion was higher in the punishment compared to the reward condition (median and [25th, 75th percentile] values: reward (before) = 24.10 [9.09, 35,06]%, punishment (before) = 36.07 [25.57, 44.86]%, z = −3.75, *p* < 0.001; reward (after) = 5.39 [0, 11.29]%, punishment (after) = 16.83 [8.02, 28.48]%, z = −4.31, *p* < 0.0001, CF 2). Thus, the higher overall number of switches in the punishment condition was not restricted to exploring all choice options, but continued to be increased after successful learning. Note that because the number of trials before and after reaching the criterion varied across participants and conditions, we used the relative number of switches (in %) for this analysis.

Analyses of reaction times further supported our hypotheses. In line with hypothesis 1, learning was accompanied by a significant decrease in reaction times (RT) over the course of the experiment [main effect of task block: *F*_(2.035, 87.507)_ = 25.488, *p* < 0.001, all pairwise differences statistically significant with *p* < 0.0016, CF 6, except for the change from block 2 to block 3, Figure 2B]. In line with hypothesis 2, we observed a main effect of valence [*F*_(1.741, 74.855)_ = 36.866, *p* < 0.0001] with longest RTs in punishment trials (854.75 ms ± 17.59), shortest RTs in reward trials (757.07 ms ± 18.05) and RTs in neutral trials (811.56 ms ± 17.51) in between (all pairwise comparisons statistically significant at *p* < 0.0001, CF 3).

Finally, changes in valence and arousal ratings before and after learning were in line with our behavioral findings, with significantly increased valence and arousal ratings for the high probability reward symbol, and significantly increased arousal ratings, but decreased valence ratings for the punishment symbols after learning (Supplementary Material). These findings corroborate the ecological validity of our task design.

### Computational modeling and analysis of learning parameters

After fitting the model to each participant's behavioral data, we first accessed model adequacy by means of the consistency parameter β. Across participants, the model parameter β explained a significant 54% of the variability in switching behavior [linear regression, *R*^2^ = 0.54, adjusted *R*^2^ = 0.53, *F*_(1, 46)_ = 53.41, *p* < 0.0001] speaking for model behavior that, after model fitting, captured significant portions of variability in participants' behavior. In addition, BIC-values obtained across participants did not significantly differ with respect to the factors gender and obesity (both *p* > 0.09). Thus, model fit was comparable across participant groups, a prerequisite for parameter comparison across groups as presented below.

From the fitted models, three independent learning rates for the reward, punishment, and neutral condition were derived for each subject. Across participants, learning rates in the reward (0.1 ± 0.07) and the punishment (0.07 ± 0.04) condition were significantly increased compared to the neutral [0.001, (0.001, 0.26)] condition (z = 3.687 and z = 3.551, respectively, both *p* < 0.0004, CF 2), again reflecting learning in both reinforcement-based conditions. In addition better learning performance in the reward compared to the punishment condition was accompanied in a small but significant difference in learning rates with a higher learning rate for reward compared to punishment trials [main effect of condition, *F*_(1, 47)_ = 4.09, *p* = 0.04]. This reflects the different speed of learning between the two conditions, as a smaller learning rate directly translates to a slower albeit correct update of value representations over the course of learning.

### Impact of personality traits and working memory capacity

In order to ensure that none of the observed behavioral effects were simply attributable to a systematic impact of the previously identified personality traits or working memory, we first analyzed potential group differences of these factors and their correlations. Detailed statistical results of this analysis are provided as Supplementary Material. Small gender differences were observed for BAS reward responsiveness, total BIS score, UPPS urgency, UPPS perseverance, and the WMS-R score. Bivariate correlations between these scores and performance measures did not reach significance. Thus, none of the observed behavioral effects were merely reflecting differences in personality traits or working memory capacity. Consequently, we omitted these factors in the subsequent analysis of phasic IBI changes in response to the presentation of stimuli and feedback.

### Analysis of autonomic responses

**Figure 3:**
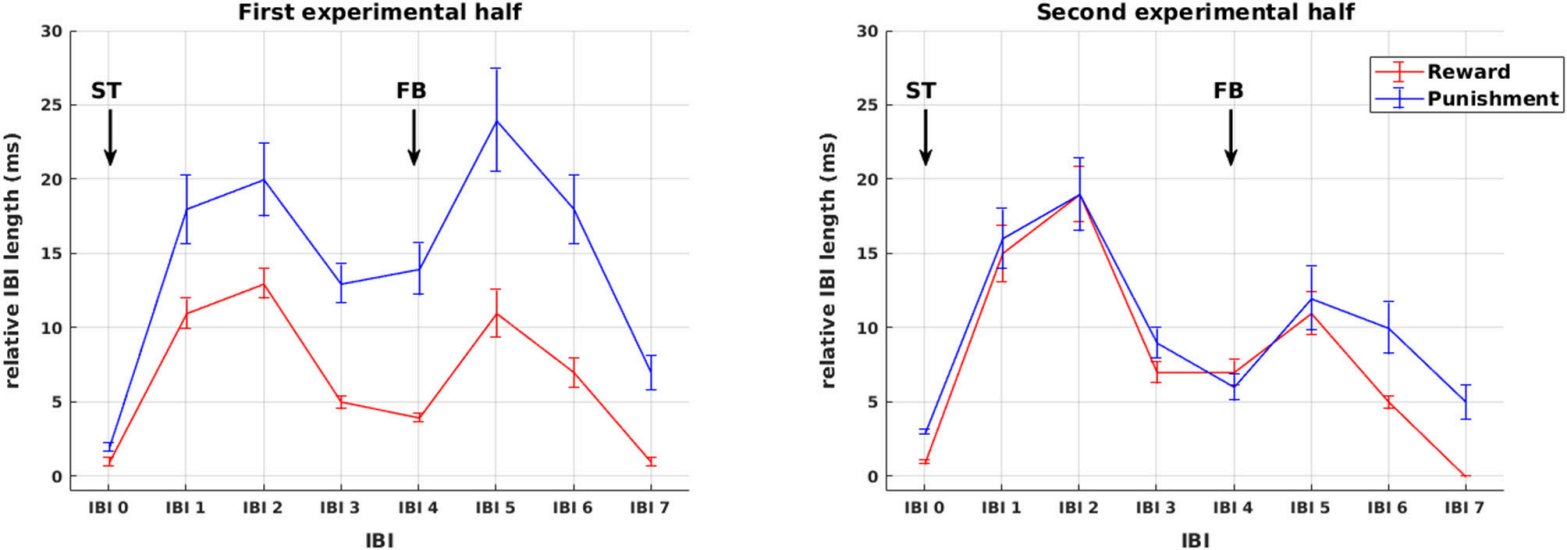
Phasic cardiac responses. Sequence of IBIs in response to reward (red) and punishment (blue) for the first **(left)** and the second **(right)** experimental half. For the purpose of plotting responses to stimuli and feedback on a common scale, in this figure all IBIs from stimulus presentation to HR recovery after feedback presentation are referenced to a common IBI −2 prior to stimulus presentation and named in relation to stimulus presentation IBI 0 to IBI 7. Arrows mark the presentation of stimuli (ST) and feedback (FB). Note that the statistical analysis of IBIs was performed separately for stimulus and feedback presentation (see Figures 4, 5).

Sequences of mean IBIs from stimulus presentation to HR recovery after feedback presentation are shown in Figure 3. Across all participants, changes in IBI length are plotted separately for reward and punishment trials during the first and second experimental half. As becomes obvious by visual inspection already, in the first experimental half, IBI deceleration was stronger in the punishment compared to the reward condition for both the presentation of stimuli as well as feedback. These differences vanished later in the experiment. In order to disentangle the impact of stimulus and feedback presentation on IBI length, changes in IBI length were assessed in the following detailed statistical analyses independently for the presentation of stimuli and the presentation of feedback.

**Figure 4:**
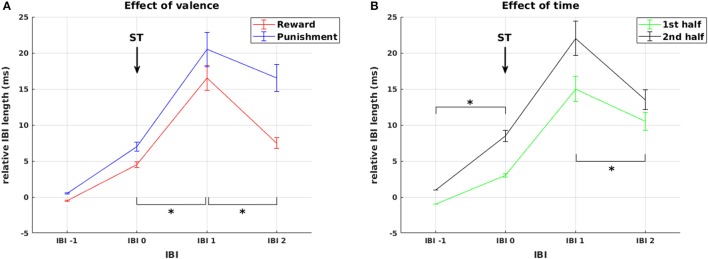
Cardiac responses to stimulus presentation. Effects of valence and time on changes in relative IBI length around stimulus presentation. **(A)** Deceleration in response to stimulus presentation was stronger and prolonged for stimuli predicting punishment compared to reward. **(B)** Cardiac reactivity in response to ST presentation was more pronounced during the second experimental half. Arrows mark the presentation of stimuli (ST). Significant differences (at *p* < 0.05) between conditions or experimental half 1 and 2 in the slope between neighboring IBIs are marked with ^*^.

First, we statistically analyzed mean IBI differences in response to **stimulus** presentation. In addition to the main effect of IBI [*F*_(1.68, 74.06)_ = 29.66, *p* < 0.0001], we observed a main effect of valence [*F*_(1, 44)_ = 12.44, *p* = 0.001] and a significant IBI × valence interaction [*F*_(2.38, 104.61)_ = 23.12, *p* < 0.0001, Figure 4A]. This was driven by a significantly higher increase in IBI length from IBI 0 to IBI 1 in punishment trials compared to reward trials [*F*_(1, 44)_ = 4.50, *p* = 0.040], representing stronger initial deceleration in response to stimuli predicting potential punishment. This was followed by a smaller decrease in IBI length from IBI 1 to IBI 2 in the punishment condition [*F*_(1, 44)_ = 27.01, *p* < 0.0001], representing a pronounced prolonged deceleration in response to stimuli predicting potential punishment. Thus, HR changes in response to stimulus presentation followed the pattern that we predicted in hypothesis 3 for HR responses to feedback, with pronounced reactivity for punishment compared to reward.

Additionally, we observed a main effect of experimental half [*F*_(1, 44)_ = 10.30, *p* = 0.002] and a significant IBI × experimental half interaction [*F*_(2.3, 100.99)_ = 5.50, *p* = 0.004, Figure 4B]. This interaction resulted from higher increase in IBI length from IBI −1 to IBI 0 [*F*_(1, 44)_ = 10.86, *p* = 0.002] and a stronger decrease from IBI 1 to IBI 2 [*F*_(1, 44)_ = 9.57, *p* = 0.003] in the second compared to the first half of the experiment. Thus, across reward and punishment conditions, anticipatory deceleration to the stimulus and recovery after stimulus presentation increased significantly over the course of the experiment. This already points at a shift in HR responsiveness over time, which is more directly addressed below.

Second, we analyzed mean IBI differences in response to **feedback**. The analysis revealed a main effect of IBI [*F*_(2.05, 89.97)_ = 9.07, *p* < 0.0001] and a significant three-way interaction of experimental half × IBI × valence [*F*_(2.56, 112.51)_ = 4.453, *p* = 0.008], again signifying an impact of the factors valence and time on IBI. However, as we also found a significant four-way interaction experimental half × IBI × valence × gender [*F*_(2.56, 112.51)_ = 2.90, *p* = 0.046], the impact of valence and time cannot be interpreted without considering the factor gender.

**Figure 5:**
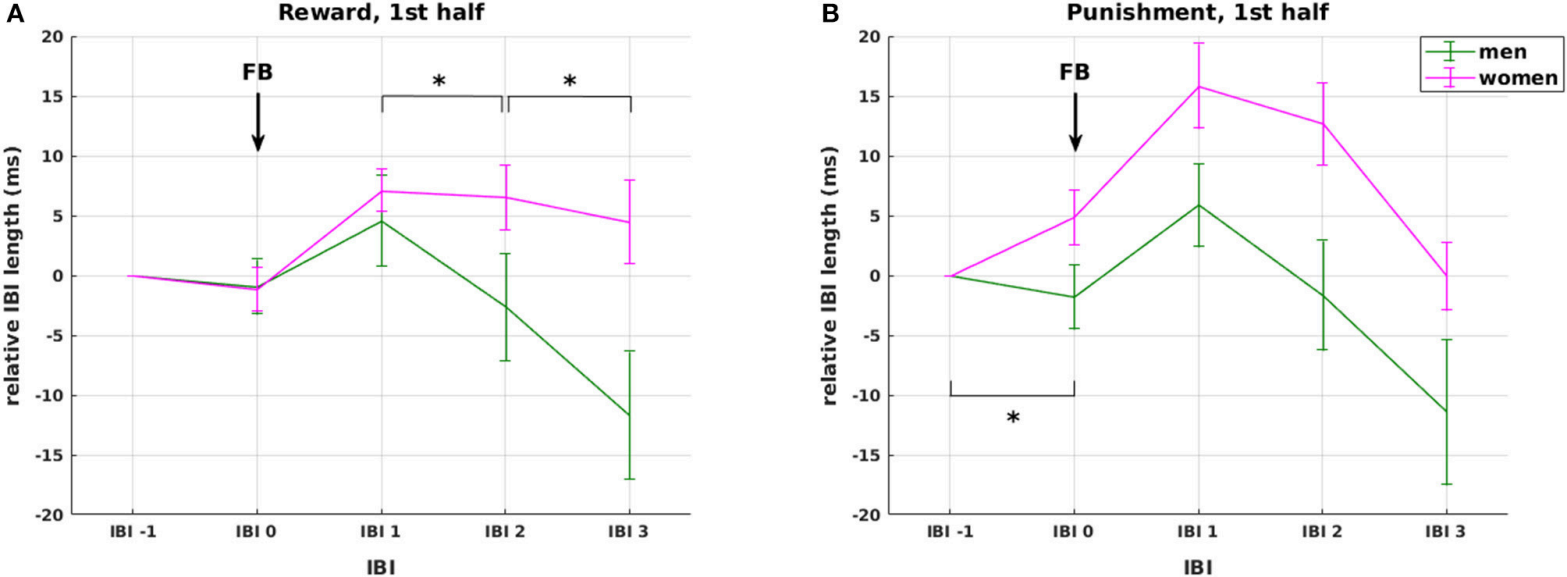
Effects of weight status and gender. Interaction between gender and valence on changes in relative IBI length around feedback presentation during the first experimental half. The interaction was driven by (1) stronger overall cardiac reactivity to feedback presentation in women compared to men (red vs. green lines), and (2) stronger anticipatory deceleration and faster recovery in the punishment **(B)** compared to the reward condition **(A)** in women only with no observable differences in men. None of these effects was observable in the second half of the experiment. Arrows mark the presentation of feedback (FB). Significant differences (at *p* < 0.05) between genders in the slope between neighboring IBIs are marked with ^*^.

Regarding the factor valence, our analysis shows that IBI 0, IBI 1, IBI 2 were significantly longer in the punishment compared to the reward condition, but only in women (*F*_(1, 44)_ = 6.157, *p* = 0.017; *F*_(1, 44)_ = 8.931, *p* = 0.005; *F*_(1, 44)_ = 4.253, *p* = 0.045, respectively, Figure 5 red lines), not in men (all *p* > 0.66, Figure 5 green lines). The difference between reward and punishment in women was driven by higher anticipatory deceleration from IBI −1 to IBI 0 in punishment compared to reward trials [*t*_(23)_ = 2.28, *p* = 0.033], and a prolonged deceleration from IBI 2 to IBI 3 in the reward compared to the punishment condition [*t*_(23)_ = 4.97, *p* < 0.0001]. Thus, while these findings support our hypothesis 3 of a significant HR deceleration in response to negative feedback, the expected differential effect of positive and negative feedback was gender specific. Importantly, the effects of feedback valence on IBI were significant only in the first, but not in the second experimental half, which again points at a shift in HR responsiveness over time.

To directly address the hypothesized **shift in HR responsiveness** from feedback to stimulus presentation during learning, we analyzed for each subject the mean area under the curve (AUC) of changes in IBI length following stimulus and feedback presentation. Most importantly, we observed an event type (stimulus/feedback presentation) × experimental half interaction [*F*_(1, 44)_ = 12.97, *p* = 0.001] which was driven by significantly higher responses to stimulus presentation compared to feedback presentation in the second half of the experiment [*F*_(1, 44)_ = 8.70, *p* = 0.005]. This strongly supports our hypothesis 4 as it represents the expected shift from relying on external feedback to update choice behavior to an internal monitoring system evaluating acquired knowledge in the course of learning.

Addressing our final hypothesis, we assessed the relationship between IBI length and strength of PE signal after the presentation of positive or negative feedback. This relationship was modeled by a GLM with subsequent statistical analysis of the GLM coefficients. Across subjects, all GLM coefficients corresponding to IBI 0, IBI 1, and IBI 2 after feedback presentation significantly differed from zero [all *t*_(47)_ > 5.89, all *p* < 0.001, CF 6]. Thus, across subjects the three IBIs following positive or negative feedback were systematically correlated with the strength of the PE signal.

Comparing standardized coefficients across IBIs and conditions revealed a main effect of valence [*F*_(1, 47)_ = 6.21, *p* = 0.016] with higher mean coefficients in the punishment (0.516 ± 0.055) compared to the reward condition (0.386 ± 0.036), and a main effect of IBI [*F*_(2, 94)_ = 8.28, *p* < 0.001] with a significantly higher coefficient for IBI 1 (0.552 ± 0.060) compared to IBI 0 (0.369 ± 0.0034) and compared to IBI 2 (0.432 ± 0.042, *p* = 0.0003 and *p* = 0.008, respectively, CF 3). In line with our hypothesis, these results show that phasic changes in IBI following feedback processing, in particular changes in IBI 1, directly reflect the strength of PE signals, i.e., the degree of discrepancy between expected and actual outcome of an action. This relationship is particularly pronounced for negative feedback.

### The effects of weight status and gender

Weight status and gender influenced some but not all investigated aspects of reinforcement learning and feedback processing both on the behavioral and the physiological level. For the sake of succinctness, we summarize below all significant effects of these two factors in the different analyses including corresponding *p*-values. Detailed statistical analyses of these effects can be found in the Supplementary Material.

On the **behavioral level**, we observed a significant condition-specific impact of weight status on learning speed. In the punishment condition, participants with obesity reached the learning criterion significantly later than lean participants (*p* = 0.036). With respect to the factor gender, we observed a difference in task performance with higher overall scores for men than in women (at a trend level *p* = 0.094) and more advantageous choices for men than women in the reward condition (*p* = 0.036, Figure 2C). This was accompanied by a significantly higher number of switches in women than men in both reward (*p* = 0.023) and punishment trials (*p* = 0.045, Figure 2D). Most importantly, in the reward condition women more often than men continued to switch between choices *after* reaching the learning criterion (*p* = 0.007), leading to reduced performance in learning from reward in women. In contrast, learning rates derived from the computational model did not differ between genders (*p* = 0.80). Thus, the observed performance differences between genders were not rooted in differential integration of new experiences into existing knowledge, but rather in the inconsistency of choice behavior as reflected in the increased switching in women even after successful learning.

On the **autonomic level**, we observed a three-way interaction of IBI with obesity and gender in the phasic cardiac responses to stimulus presentation (*p* = 0.039). This interaction was driven by an increased initial deceleration in response to stimulus presentation in lean men. We further observed gender differences in cardiac responses to positive and negative feedback presentation during the first experimental half. In reward trials, women showed slower HR recovery than men with smaller HR deceleration from IBI 1 to IBI 2 and from IBI 2 to IBI 3 (*p* = 0.015 and *p* = 0.024, respectively, Figure 5A). In the punishment condition, women showed overall increased HR responses compared to men, caused by a stronger anticipatory deceleration from IBI −1 to IBI 0 (*p* = 0.006, Figure 5B).

Finally, a three-way interaction of experimental half, gender, and obesity was observed in AUC-values (*p* = 0.024) with significantly higher responses in lean men compared to lean women during the first experimental half (*p* = 0.022). This speaks for a faster internalization of stimulus-outcome associations in lean men during the initial phase of learning.

## Discussion

Successful learning and behavioral adaptation hinges on the sufficient detection and adequate evaluation of external feedback and several studies have established a link between the processing of external feedback and autonomic reactions (e.g., Somsen et al., 2000; Crone et al., 2003, 2005; Groen et al., 2007). Using a probabilistic learning task, we investigated the cardiac concomitants of reinforcement-based learning and the impact of weight status and gender on learning performance. Further, we introduced a new method for simultaneously analyzing behavioral and autonomic data that enabled us to link these two modalities on a trial-by-trial basis. Our study makes several important contributions to our understanding of reinforcement learning and related autonomic reactions. We could show that learning and feedback processing is closely mirrored by phasic cardiac responses on several levels (1) On a trial-by-trial basis phasic cardiac responses after feedback are correlated with the strength of PE signals that encode the deviation between expected and actual outcome of a choice or action. (2) Cardiac responses shifted from feedback presentation at the beginning of learning to stimulus presentation at later stages. (3) Feedback valence impacted on cardiac responses with faster and prolonged HR deceleration in response to negative feedback. Additionally, we observed differential impacts of weight-status and gender on both learning performance and changes in HR responses. In the following, we discuss these results in more detail.

Several previous studies have shown that during reinforcement-based learning, the processing of feedback is reflected in HR slowing. However, investigations into the precise meaning of the observed effects yielded heterogeneous results so far. Van Der Veen et al. (2004) reported that cardiac slowing was stronger and prolonged for negative compared to positive feedback, but did not discriminate between informative and non-informative feedback. They argued that HR deceleration may thus be sensitive to the valence rather than relevance of feedback. Others found that cardiac responses were stronger toward unexpected feedback and suggested that this reflects the monitoring of learning-relevant information (Somsen et al., 2000; Crone et al., 2003, 2005). With our approach we were able to directly address these different views, linking learning performance and behavioral adaptation to estimates of internal learning signals. If cardiac responses merely reflected feedback valence, no direct link to PE signals would be expected. In line with previous studies, our results show an overall stronger and prolonged HR deceleration in response to punishment compared to reward. However, we additionally found that the strength of HR deceleration following feedback was indeed predictive of the strength of the model-derived PEs that indicate how much the provided feedback deviated from the participants' expectations. Interestingly, this relationship was particularly pronounced for negative feedback, which signals the need for behavioral adjustments, while positive feedback reinsures the learner that the current choice behavior is correct.

Supporting our hypotheses, we further observed that HR responses toward feedback changed over the course of learning. Specifically, each symbol pair in the current task was exclusively associated with the prospect of a reward, threat of punishment, or financially neutral feedback and these associations did not change over time. Consequently, after an initial learning phase the participants should have been able to anticipate the potential trial outcome associated with a presented symbol pair. Indeed, this was reflected in HR responses, showing a shift of cardiac responses from the presentation of feedback during the first half of the experiment to the presentation of the symbol pairs in the second half. This corroborates findings by Groen et al. (2007), who observed a general reduction in feedback-related HR deceleration with learning, together with a shift of HR slowing from the IBI following feedback presentation to the IBI preceding feedback presentation in later stages of the experiment.

Taken together, these results provide strong new evidence for the assumption that HR deceleration during learning is sensitive to learning-relevant information and reflects an internal monitoring system to detect the violation of expectations derived from preceding experience, while over the course of learning a shift from the dependency on external feedback signals at initial stages to the use of internal error detection mechanisms occurs.

Successful feedback-based learning requires the integration of multiple processes. In each trial of a reinforcement learning paradigm, the learner needs to monitor incoming feedback, build and maintain value representations, construct a PE signal, update value representations according to the PE and, when necessary, adjust behavior for future actions and decisions. Deficits in feedback-based learning can be caused by impairments in any of these sub-processes or a combination thereof. For example, phenomenologically similar deficits in reinforcement learning can be observed in young children and older adults, but these deficits are likely attributable to impairments in different underlying mechanisms: a reduced executive control capacity in children, and a decline in the ability for differentiated value representation with age (Hämmerer and Eppinger, 2012). However, based on behavioral observations alone, the various aspects of the learning process cannot clearly be disentangled.

Our behavioral data combined with computational modeling allowed us to partly disentangle the sub-processes of reinforcement learning in a within-subject fashion. In line with our hypothesis, learning was evident on the behavioral level from increasing overall task scores, increasing numbers of optimal choices, and decreasing reaction times over time across participants. In addition, participants changed their valence and arousal ratings for the symbols according to their probabilities of predicting reward or punishment. Together, these results signify behavioral adaptations that were generally appropriate for the task at hand. Further, higher model-derived learning rates in the reward and punishment compared to the neutral condition point at appropriate updating of value representations in both conditions. However, we also observed systematic differences between the processing of positive and negative feedback with more advantageous choices and shorter reaction times for reward than for punishment. Computational modeling revealed a small difference in learning rate between the reward and punishment condition. This was corroborated by the fact that participants on average needed longer to reach the learning criterion in the punishment than in the reward condition. Both results indicate a slower updating of value representations after negative feedback. In addition, reduced performance in the punishment condition could be linked to switching behavior with more switching after negative than after positive feedback. While this speaks for an appropriate behavioral adaptation after punishment, increased switching was also observable after the learning criterion was reached, i.e., after participants should have learned that it is advantageous to stick to a certain symbol, even if it is occasionally punished.

In sum, behavioral analysis and computational modeling suggest that the observed differences in task performance when learning from reward and learning from punishment were not caused by insufficient sensitivity to or internal representation of negative feedback. Rather they are attributable to (1) a difference in value updating or, in other words, different speed of learning between these conditions and (2) differences in behavioral adaptation in the exploration and exploitation phase of learning with continued increased switching after successful learning in the punishment condition. While our analyses cannot provide a full explanation of differences in positive and negative feedback, they would predict similar value representations and PE signals on the neural level, while the utilization of these signals for learning might differ. This hypothesis will be subject of our future investigations.

In addition to general psychophysiological correlates of reinforcement-based learning, we hypothesized that weight status and gender might impact on performance and HR responses for rewards and punishments. Supporting our hypotheses, we found that individuals with obesity showed a slower learning of advantageous choice behavior in the punishment learning condition. Specifically, they needed more time to learn to stably choose the advantageous choice option. This is in line with previous reports of a compromised learning performance in individuals with obesity (Coppin et al., 2014). Interestingly, in the current study, weight status was not related to other performance measures that captured behavior across the whole experiment (e.g., learning rate, number of advantageous choices). This suggests that differences were restricted to the initial learning phase, while individuals with obesity were able to compensate and reach a comparable performance across the whole experiment. Indeed, using the same paradigm in a functional magnetic resonance study, we found obesity-related impairments particularly during the first half of the experiment (Kube et al., submitted). In the same vein, Zhang et al. (2014) reported differences in learning performance between lean and obese women within as few as ~20 trials, supporting the idea of an early acquisition deficit in obesity. Various mechanisms for impaired reinforcement-based learning have been identified in other populations, showing alterations in PE encoding in aging (Eppinger et al., 2013), PE utilization in addiction (Park et al., 2010), and working memory capacity in healthy individuals (Collins and Frank, 2012) to be related to a poorer performance. Indeed, in a recent paper, Collins et al. (2017) argue that learning in simple reinforcement-based tasks is best explained by a mixture of working memory and PE processes. Though we have not found group differences in visual working memory in the current study, obesity-related impairments in other measures of working memory capacity have been shown to affect preference learning (Coppin et al., 2014). Additionally, we have previously linked reinforcement learning deficits in obesity to a less efficient utilization of negative PEs in the striatum (Mathar et al., 2017a), thus adding another potential mechanism to explain obesity-related effects in the current study. In sum, these results suggest that individuals with obesity exhibit a slower learning performance. However, so far, the underlying mechanisms have not been fully discovered, with potentially different mechanisms interacting to explain learning alterations in individuals with obesity.

Complementary to the effect of weight-status, we found a modulation of learning performance and HR responses by gender. In line with numerous previous studies (e.g., Crone et al., 2003; Groen et al., 2007), cardiac responses to external stimuli were characterized by an initial HR deceleration, followed by an acceleratory recovery response. However, in our study, women compared to men exhibited a prolonged HR deceleration after rewards and a stronger initial deceleration to punishment particularly during the first half of the experiment. As detailed above, previous studies have mostly reported a stronger and prolonged deceleration to the presentation of negative stimuli (Crone et al., 2004a; Van Der Veen et al., 2004), but in the context of learning stronger HR responses may likewise be associated to the processing of learning-relevant information in general (Somsen et al., 2000; Crone et al., 2003, 2005). Further, responses may also depend on the motivational significance of the stimulus material. For instance, a stronger deceleration seems to occur for large compared to small monetary losses (Crone et al., 2004b), while highly arousing positive and negative pictures have been found to elicit stronger cardiac deceleration than low arousing emotional stimuli (Balconi et al., 2009). Consequently, stronger HR responses to both reward and punishment in women compared to men could speak for a stronger utilization of learning-relevant information or a generally heightened sensitivity to feedback stimuli in women.

Surprisingly, this was not directly mirrored in the learning indices, as women exhibited a poorer performance than men, particularly when learning from reward feedback. Gender-related influences on performance in reward-based choice tasks have been frequently reported in other studies. For instance, men have been shown to exhibit a higher performance in reversal learning tasks than women (Evans and Hampson, 2015) and likewise outperform women in the Iowa Gambling task (Weller et al., 2009; Evans and Hampson, 2015). Interestingly, this seems to be driven by the fact that men quickly learn to choose cards from decks associated with smaller immediate rewards, but a larger net payoff across trials, while women keep choosing from a deck with frequent high immediate rewards and even higher, but infrequent losses (Overman, 2004). Males thus appear to be more sensitive to the long-term monetary outcomes of the task, and females are more sensitive to immediate rewards. Indeed, in our study, women were characterized by higher trait reward sensitivity than men. Likewise, stronger HR responses to feedback may be an indicator of heightened feedback sensitivity in women. Interestingly, an allegedly lower learning performance in women than men was accompanied by comparable learning rates, speaking for similar value updating processes in both genders and against insufficient feedback monitoring in women, respectively. Instead, women showed more inconsistent choice behavior, i.e., even after they had stably learned to choose the more advantageous symbol in reward trials, they more often switched to the other (disadvantageous) symbol than men. In the light of previous studies, this could indicate that despite their knowledge of the advantageous choice options, women were more susceptible to the presentation of probabilistic (misleading) feedback, i.e., the infrequent omission of an expected reward after an advantageous choice and the infrequent receipt of a reward after a disadvantageous choice may have fostered switching behavior more strongly in women than men. In sum, our results suggest that the observed performance deficits in learning from reward in women were not caused by deficits in feedback monitoring or the representation and updating of stimulus values, but by a higher responsiveness to reinforcement that was accompanied by more pronounced HR responses and interfered with the beneficial behavior in the current study.

Lastly, we found evidence for a combined influence of obesity and gender on HR responses. Specifically, lean men exhibited a stronger initial deceleration during stimulus presentation than lean women, suggesting a stronger anticipatory response to the prospect of reinforcement. However, HR changes did not translate to alterations in behavioral performance and no differences were found between men and women with obesity. This is a clearly surprising finding, especially, since previous studies have highlighted that alterations in executive functioning and behavioral adaptation may be particularly pronounced in women with obesity, while performance of men with obesity remains relatively intact (Weller et al., 2008; Horstmann et al., 2011; Zhang et al., 2014). Consequently, the influence of gender on obesity-effects seems specific for certain types of stimuli and tasks and requires further consolidation.

Finally, some limitations of the current study design must be acknowledged: First, it has been shown that cardiac markers are significantly influenced by stimulus timing during cognitive and emotional processing. For instance, negative emotional stimuli presented at systole are detected more easily (Garfinkel et al., 2014) and perceived as more intense than stimuli presented at diastole (Gray et al., 2012; Garfinkel et al., 2014), while words encoded at systole are less well remembered than words encoded at diastole (Garfinkel et al., 2013). In the current study, stimulus and feedback presentation were not time-locked to the onset of systole or diastole. Instead they were presented at variable points within the cardiac cycle. This leaves the possibility that differences in cardiac responses may have been affected by incidental differences in stimulus timing within the cardiac cycle. However, trial order was pseudo-randomized and trials were separated by varying ITI lengths and delay periods, thus rendering an influence of stimulus onset unlikely. Second, the interval between stimulus and feedback presentation was relatively small with a maximum of 3,500 ms. While this is sufficient to minimize the impact of the strongest autonomic reactions to the stimulus (IBI 0 and IBI 1) on the following feedback presentation, a full recovery of cardiac responses before feedback presentation is unlikely. It would clearly be ideal to separate both phases by longer delay periods to await a recovery to baseline before feedback presentation. However, this would in turn result in significantly longer trials and a significantly increased duration of the experiment, which can potentially facilitate fatigue and decreases attention toward the task. Instead, we used an IBI *after* stimulus presentation as reference for the feedback-related IBIs, thus technically excluding any stimulus-related carryover effects from stimulus to feedback presentation. Similarly, feedback was followed by the next trial's stimulus presentation after 3,600 to 4,200 ms which again might not have been entirely sufficient for full recovery. To alleviate this problem, we used jittered ITIs and a pseudo-randomized trial order, and statistically ensured that the reference IBIs for the stimulus analyses were independent of all factors that could impact on the subsequent IBIs. Third, we did not measure respiration in the current study, though it has been shown that HR fluctuates depending on respiration. Heart periods become shorter or longer in phase relationship with inspiration and expiration (Berntson et al., 1993). While some highlight the need to remove respiratory influences from the ECG signal (Quintana and Heathers, 2014), others argue that resting HR and respiration share a common basis (Thayer et al., 2011). Thus, under spontaneous breathing conditions, controlling for respiration may remove variance in the ECG signal that the researcher is actually interested in (Laborde et al., 2017). Nevertheless, measuring respiration simultaneously to ECG could have helped to detect non-cyclical breathing patterns (e.g. sighs) that could bias HR results (Vaschillo et al., 2015). Lastly, it would have been interesting to investigate in more detail a potential link between the stimulus—and feedback-related cardiac responses during learning with the observed differences in switching behavior in the reward and punishment condition and between genders, in particular after reaching the learning criterion. Unfortunately, our task design did not allow for such a detailed analysis as the number of switch trials after successful learning were too small for a statistically sound analysis. An experimental design that provokes higher switching rates and includes conditions where switching might also be an advantageous strategy together with ECG measurements could be an interesting approach for future work to answer this question.

## Conclusion

In the present study, we investigated learning performance and cardiac concomitants of reinforcement learning together with the impact of feedback valence, gender, and weight status on learning performance and autonomic responses. We could show that the strength of cardiac responses to learning-related feedback directly reflects the strength of PE signals that alert the learner to the necessity for value updating and behavioral adaptation. Thus, phasic changes in HR seem to express processes of an internal feedback monitoring system that is sensitive to the violation of performance-based expectations. Moreover, apparent gender-related deficits in reinforcement learning were not caused by deficiencies in knowledge acquisition, but by insufficient adaptation in an environment that requires consistent choice behavior. Finally, our study adds evidence to the notion that individuals with obesity might be impaired in learning to avoid negative outcomes.

## Author contributions

LK, JK, AV, and JN conceived of the study and designed it. LK and JK performed the measurements. LK, JK, and JN analyzed the data. LK, JK, AV, and JN wrote the manuscript.

### Conflict of interest statement

The authors declare that the research was conducted in the absence of any commercial or financial relationships that could be construed as a potential conflict of interest.
