# Supplementary material for: Cardiac Concomitants of Feedback and Prediction Error Processing in Reinforcement Learning
Source: Front Neurosci. 2017 Oct 30;11:598. doi: 10.3389/fnins.2017.00598 (PMC5670147; doi:10.3389/fnins.2017.00598)
Supplement: Supplementary file 1 [file DataSheet1.docx]

Supplementary Material

Cardiac concomitants of feedback and prediction error processing in reinforcement learning

Lucas Kastner, Jana Kube, Arno Villringer, Jane Neumann

*** Correspondence:** Corresponding Author: Jane Neumann ([neumann@cbs.mpg.de](mailto:neumann@cbs.mpg.de))

# Supplementary Data

***Analysis of HRV***

ECG was recorded during a 5 min resting period before the start of the experiment to probe baseline differences in HRV. HRV at baseline was analyzed in the time and in the frequency domain using Kubios (Version 2.2, Biosignal Analysis and Medical Imaging Group, University of Eastern Finland, http://kubios.uef.fi/). In the time domain, we calculated the mean and standard deviation of the normal-to-normal (NN) intervals (mean NN and SDNN), the percentage of NN intervals that differed from the previous interval by more than 50ms (pNN50), and the square root of squared successive differences in NN intervals (rMSSD).

In the frequency domain, we assessed total spectral power, covering a frequency band between 0 and 1 Hz, the spectral power in the high-frequency band between 0.15 and 0.40 Hz (HF) and in the low-frequency band between 0.04 and 0.15 Hz (LF) as well as the low frequency/high frequency ratio. HF and LF power were additionally transformed to normalized values. These values emphasize the controlled and balanced behavior of the two branches of the autonomic nervous system and minimize the effect of the changes in total power on the values of LF and HF components (Task Force 1996).

Measures of HRV at baseline in both the time and the frequency domain were compared by Mann-Whitney-U-Tests for the factors gender and obesity. In line with the literature, we observed significant differences between male and female participants in HF and LF (in normalized units) as well as in the HF/LF ratio with significantly higher HF values in females (43.30 [14.42, 86.22]) than in males (24.17 [4.11, 69.47], U = 425, z = 2,825 p = .005), significantly higher LF values in males (75.81 [30.53, 95.88]) than in females (56.67 [13.77, 85.56], U = 150, z = -2,846 p = .004), and significantly higher LF/HF ratio in males (3.21 [0.44, 23.32]) than in females (1.32 [0.16, 5.93], U = 151, z = -2,825 p = .005). Note that the differences in HF and LF did not reach significance when assessed in absolute values (p = 0.45 and p = 0.07, respectively). No other frequency domain or time domain measure differed significantly between male and female participants. In addition, no significant difference between lean and obese participants was observed for any obtained HRV measure.

***Analysis of Valence and Arousal Ratings***

First, we assessed possible differences in valence and arousal ratings between symbols prior to the task. As intended by the abstract design of the symbols, no differences between the symbols and no influence of gender or obesity on initial valence and arousal ratings were observed (all p > 0.33). Second, we investigated task-induced changes in the valence and arousal ratings of the individual symbols. After the task, both valence and arousal ratings for the high probability reward symbol significantly increased (valence: pre = 5.27, post = 8.00, F(44,1) = 47.16, p < 0.001; arousal: pre = 2.66, post = 3.62, F(44,1) = 8.32, p = 0.006). For both symbols in the punishment condition, valence ratings significantly decreased (high probability loss avoidance: pre = 5.45, post = 3.89, F(44,1) = 14.31, p < 0.001; low probability loss avoidance: pre = 5.70, post = 2.68, F(44,1) = 75.95, p < 0.001), while arousal ratings significantly increased (high probability loss avoidance: pre = 2.98, post = 5.12, F(44,1) = 35.85, p < 0.001; low probability loss avoidance: pre = 2.91, post = 4.96, F(44,1) = 24.97, p < 0.001). No significant changes in valence or arousal ratings were observed for the symbol with a low probability of reward, and for the symbol with high probability of feedback in the neutral condition. For the symbol with low probability of feedback in the neutral condition, arousal ratings slightly but significantly decreased (pre = 3.10, post = 2.29, F(44,1) = 9.04, p = 0.004), while no significant change was observed for the symbol’s valence. In sum, these findings corroborate the ecological validity of our task design. The factors gender and obesity had no significant influence on any of the changes in valence or arousal ratings.

***Analysis of personality traits and working memory capacity***

Responsiveness to reward and punishment was assessed by the German version of the BIS/BAS questionnaire (Carver and White, 1984, Strobel et al. 2001). With respect to the factor gender, we observed a statistically significant difference in the reward responsiveness score of the BAS (F(1,44) = 4.342, p = .043) with higher values in women (3.39±0.37) compared to men (3.17±0.37), accompanied by significantly increased total BIS score in women (2.98±0.13) compared to men (2.44±0.36; F(1,44) = 26.081, p < .0005). No significant differences were observed between participants with and without obesity.

Trait impulsivity was assessed by the German version of the UPPS Impulsive Behavior Scale (Whiteside and Lynam 2001, Schmidt et al. 2008). Regarding gender, we observed a statistically significant difference in UPPS urgency score (F(1,44) = 4.964, p = .031) with higher scores in women (27.42±6.14) compared to men (23.96±4.39). In addition, we observed a significant gender x obesity interaction in the UPPS perseverance score (F(1,44) = 6.881, p = 0.012). This interaction was driven by larger scores in lean men (21.00±3.46) compared to lean women (16.50±2.77, t(22) = 3.51, p= 0.002, CF 4), while scores in participants with obesity were comparable for men (18.17±3.38) and women (19.17±4.65) with no statistically significant difference.

Working memory capacity was assessed by the Revised Wechsler Memory Scale (WMS-R), subtest Figural Memory, German version. (Härting et al. 2000; Wechsler 1987). We observed a small but significant difference in working memory test scores between male (8.66±1.13) and female (7.91±1.10) participants (F1,44) = 5.805, p= 0.020). No significant difference in working memory capacity was observed for participants with and without obesity.

Bivariate correlations between these scores and performance measures did not reach significance (all p > 0.16).

***The effects of weight status and gender***

Weight status and gender influenced some but not all investigated aspects of reinforcement learning and feedback processing both on the behavioral and the physiological level. Here we report all significant effects of these two factors in the different statistical analyses.

Regarding weight status, we observed on the behavioral level a significant condition-specific difference in learning speed between participants with and without obesity. Specifically, in the punishment condition participants with obesity reached the learning criterion significantly later (on average after 25.5 [10, 56] trials than lean participants (on average after 18 [10, 81] trials, U = 151.0, z = -2.10, p = 0.036).

With respect to the factor gender, we observed a trend for a difference in task performance with higher scores in men than in women (F(1) = 2.93, p=.094). Further, men exhibited more advantageous choices (75.5 [38,80]) than women (69.5 [32,80], U = 186.5, z = -2.098, p = .036), however, this effect was only present in the reward condition. Lower scores and lower number of advantageous choices in women were accompanied by a significantly higher number of switches in women than men in both reward (men: 6 [0,40], women: 11 [0, 37], U = 147.0, z = 2.27, p = .023) and punishment trials (men: 16 [3, 40], women: 23 [3,44], U = 191.0, z = 2.00, p = .045). Most importantly, in the reward condition, we observed significantly higher number of switches after reaching the learning criterion in women (9.61 [0, 44.28] %) than in men (0 [0, 52.94] %, U = 127.5, z = -2.72, p = 0.007). Thus, in the reward condition, women more often than men continued to switch between choices after successful learning, leading to reduced performance in learning from reward in women. In contrast, learning rates derived from the computational model did not differ between genders (p = 0.80). Thus, the observed performance differences between genders were not rooted in differential integration of new experiences into existing knowledge, but rather in the inconsistency of choice behavior as reflected in the increased switching in women described above.

Reaction times did not differ across the factors obesity and gender, and no other interaction of gender and weight status with the analyzed behavioral factors reached significance (all p >= .175).

Gender and obesity impacted on autonomic responses in a complex and interacting way. In the analysis of phasic cardiac responses to stimulus presentation we observed a significant three-way interaction of IBI with obesity and gender (F(1.68 ,74.06) = 3.63, p = .039). This effect was carried by an obesity x gender interaction in the increase of IBI length between IBI 0 and IBI 1 (F(1,44)=4.595, p=.038). Thereby, significantly higher initial deceleration was observed in men compared to women in the lean (t(22) = 2.30, p = 0.032) but not in the obese group (p= 0.39). Further, initial deceleration was significantly higher in lean than in obese men (t(22) = 2.28, p = 0.033) but not compared to women (p= 0.56). Thus, the three-way interaction was driven by an increased initial deceleration in response to stimulus presentation in lean men.

Regarding cardiac responses to feedback presentation, with further analyzed the four-way interaction experimental half x IBI x valence x gender (F(2.56,112.51) = 2.90, p = 0.046) reported in the main manuscript with respect to the gender differences, whereby the interaction of gender and feedback valence was only present during the first experimental half. In reward trials, women showed significantly longer IBI 3 and a trend of a longer IBI 2 than men (F (1,44) = 6.66, p = 0.013; F (1,44) = 3.16, p = 0.082, respectively). This was caused by slower recovery from deceleration from IBI 1 to IBI 2 and from IBI 2 to IBI 3 in women compared to men (t(46) = 2.53, p = 0.015 and t(46) = 2.33, p = 0.024). In the punishment condition, IBI 1 and IBI 2 were significantly increased and IBI 0 was increased at a trend level in women compared to men (F (1,44) = 3.952, p = 0.043; F (1,44) = 6.01, p = 0.018; F (1,44) = 3.40, p = 0.072, respectively). This was caused by stronger anticipatory deceleration from IBI -1 to IBI 0 in women compared to men (t(46) = 1.88, p = 0.006).

Finally, the analysis of a shift in autonomic responsiveness revealed a three-way interaction of experimental half, gender, and obesity in AUC values (F(1,44) = 5.46, p = 0.024). This was driven by significantly higher responses in lean men compared to lean women (F(1,44) = 5.67, p = 0.022) in the first experimental half, with no statistical differences between men and women in the obese group. No pairwise differences were observable in the second experimental half. This speaks for a faster internalization of stimulus-outcome associations in lean men during the initial phase of learning.

***References***

Carver, C. S., & White, T. L. (1994). Behavioural inhibition, behavioural activation, and affective responses to impending reward and punishment: The BIS/BAS scales. Journal of Personality and Social Psychology, 67, 319-333.

Härting, C., Markowitsch, H.-J., Neufeld, H., Calabrese, P., Deisinger, K., & Kessler, J. (2000). Wechsler Memory Scale - Revised Edition, German Edition. Manual. Bern: Huber.

Schmidt, R. E., Gay, P., D'Acremont, M., & Van der Linden, M. (2008). A German adaptation of the UPPS impulsive behaviour scale: Psychometric properties and factor structure. Swiss Journal of Psychology, 67(2), 107-112.

Strobel, A., Beauducel, A., Debener, S., & Brocke, B. (2001). Eine deutschsprachige Version des BIS/BAS-Fragebogens von Carver und White. Zeitschrift für Differentielle und Diagnostische Psychologie, 22(3), 216–227.

Task Force of the European Society of Cardiology, and the North American Society of Pacing and Electrophysiology. (1996) Heart rate variability. Standards of measurement, physiological interpretation, and clinical use. Circulation 93: 1043–65.

Wechsler, D. (1987). Wechsler Memory Scale—Revised manual. San Antonio, TX: Psychological Corporation.

Whiteside, S. P., & Lynam, D. R. (2001). The Five Factor Model and impulsivity: using a structural model of personality to understand impulsivity. Personality and Individual Differences, 30(4), 669-689.
